# *Helicobacter pylori*: Have potential benefits been overlooked?

**DOI:** 10.1002/jgh3.12842

**Published:** 2022-11-16

**Authors:** Jonathon P Schubert, Christopher K Rayner, Samuel P Costello, Ian C Roberts‐Thomson, Samuel C Forster, Robert V Bryant

**Affiliations:** ^1^ Faculty of Health and Medical Sciences University of Adelaide Adelaide Australia; ^2^ Department of Gastroenterology The Queen Elizabeth Hospital Woodville Australia; ^3^ Department of Gastroenterology and Hepatology Royal Adelaide Hospital Adelaide Australia; ^4^ Microbiota and Systems Biology Hudson Institute of Medical Research Clayton Australia

*Helicobacter pylori (H. pylori)* has co‐evolved with humans over tens of thousands of years.^1^ While its role in the pathogenesis of peptic ulcer and gastric cancer is widely acknowledged, less publicized is the multi‐faceted physiological function that *H. pylori* plays as a commensal in the human gastric microbiome. Recent evidence has highlighted the role of *H. pylori* as an immune modulator with increasing evidence of an inverse association between *H. pylori* colonization and immune‐mediated disorders including asthma and inflammatory bowel disease (IBD). In addition, there is burgeoning evidence to suggest that *H. pylori* modulates satiety hormones, including leptin and ghrelin, that may influence appetite and contribute to weight control. Yet another issue is the relationship of *H. pylori* to gastric acid secretion, gastroesophageal reflux disease [GERD] and the rising incidence of adenocarcinoma of the lower esophagus.

The role of *H. pylori* in chronic gastric inflammation is well established. Almost all patients infected with *H. pylori* exhibit histological chronic active inflammation, even those who are asymptomatic.^2^ The association of *H. pylori* with peptic ulceration, particularly duodenal ulceration, is also clear. Whereas peptic ulceration was estimated to affect at least 10% of the human population in the mid‐20th century, current prevalence rates have substantially decreased because of falling rates of *H. pylori* infection, eradication regimens, fewer smokers, and the widespread use of medication to reduce gastric acid secretion. Gastric cancer is mostly associated with *H. pylori* [80%–90%] and will develop in approximately 1% of infected individuals over their lifetime.^3^, ^4^ However, the risk of gastric malignancy varies widely between different populations with a greater incidence of *H. pylori*‐associated cancer in Eastern and Central Asia [approximately 30 per 100 000 males], compared to regions of North and East Africa [approximately 5 per 100 000 males].^5^ Gastric mucosa‐associated lymphoid tissue [MALT] lymphoma is a rare disease but is almost always associated with *H. pylori* and may resolve with eradication of the infection.^4^

The 2015 Kyoto global consensus report on *H. pylori* gastritis recommends that all patients with this condition should receive eradication therapy, regardless of the presence of peptic ulcer or the background risk of gastric cancer.^6^ This recommendation promotes the belief that “the only good *H. pylori* is a dead *H. pylori*” and includes the assumption that the infection is associated with few, if any, beneficial effects. With the passage of time, this recommendation may need to be re‐examined in the light of new epidemiological data that suggests a relationship between *H. pylori* infection and lower risks for obesity and important gastrointestinal diseases such as IBD, GERD, and esophageal cancer.

Arguably, the management of obesity and its complications poses the greatest challenge to health care in the current era. Clearly, the most important risk factors are related to diet and lifestyle. Whether *H. pylori* infection is associated with lower body weight is less clear but appears to apply in some lower‐income countries.^7^ This effect may be related to changes in the intestinal microbiome or to the effects of gastric infection on levels of appetite‐related hormones such as leptin and ghrelin. Although the main source of leptin is adipose tissue, leptin is also produced by gastric chief and parietal cells and released in response to meals and hormonal signals.^8^, ^9^ Leptin signals satiety to the hypothalamus and is followed by diverse effects including increased energy expenditure and reduced gastric acid secretion.^10^ In contrast, ghrelin is released from oxyntic cells during fasting and reduces energy expenditure while increasing appetite and gastric acid secretion.^11^, ^12^

In individuals colonized with *H. pylori*, leptin levels are higher than in uninfected controls. Conversely, ghrelin levels substantially increase with eradication of *H. pylori*. Both effects have the potential to stimulate hunger, adipose tissue deposition, and growth hormone release.^13^ This is supported by clinical studies showing slower weight gain in infected compared to uninfected children and weight gain after the eradication of *H. pylori*.^13^, ^14^ Whether these metabolic effects of *H. pylori* are beneficial in low socioeconomic areas with marginal food availability remains unclear.^7^ However, rising rates of obesity in developed countries [where the *H. pylori* prevalence is falling], support the possibility of a link between *H. pylori* and weight control, perhaps mediated by hormonal factors.

Several studies have shown a lower‐than‐expected frequency of *H. pylori* in patients with a variety of allergic disorders such as asthma and allergic rhinitis and immunologic disorders such as IBD. One possibility is that *H. pylori* has a specific effect on immune tolerance while another is that the presence of *H. pylori* is a non‐specific marker of exposure to a more contaminated environment. The latter is widely recognized as the “hygiene hypothesis.” Recent meta‐analyses indicate that *H. pylori* colonization reduces the risk of IBD by 38%–52%.^15^, ^16^ This may be related to an effect of *H. pylori* on systemic immune homeostasis with the induction of tolerant dendritic cells and immunosuppressive regulatory T cells. These ideas are supported by murine models of colitis showing suppression of systemic inflammation in the presence of *H. pylori* infection.^17^ Whether the beneficial effects of *H. pylori* are lost after eradication of the infection remain unclear. Of interest was a recent study showing that regulatory T cells induced by *H. pylori* were able to skew the adaptive immune response towards immune tolerance with effects on T cell responses to other allergens and auto‐allergens.^18^ These effects have the potential to reduce the risk of asthma and other allergic disorders.

For clinical gastroenterologists, the final potential benefit of *H. pylori* lies in the links between gastric infection with *H. pylori*, GERD, Barrett's esophagus, and the risk of esophageal adenocarcinoma. In several countries, increasing rates of reflux oesophagitis at upper GI endoscopy have been accompanied by decreasing rates of positive urease tests.^19^ In Singapore [with a higher prevalence of *H. pylori]*, the frequency of reflux oesophagitis [3.3%] is less than one‐third of that observed in the USA.^20^ Furthermore, there is now persuasive evidence that eradication of *H. pylori* is followed by an increase in the frequency of both reflux symptoms and oesophagitis at endoscopy.^19^ For esophageal adenocarcinoma in the USA, rates have increased more than three‐fold in the last 50 years, making it the fastest rising malignancy in that country.^21^ An increase of this magnitude has not been observed in Singapore.^20^ A prospective study of the influence of *H. pylori* on Barrett's esophagus and esophageal cancer found that patients infected with *H. pylori* had a lower frequency of both Barrett's high‐grade dysplasia and Barrett's adenocarcinoma.^22^ These conclusions have been supported by other studies.^23^

Reasons for the above observations appear to rely on the complex relationship between *H. pylori* and the secretion of gastric acid. In the majority of infected individuals, gastric acid secretion is lower than in uninfected controls, presumably because of gastritis [sometimes associated with atrophic gastritis] involving the body of the stomach. In this group, eradication of infection is usually associated with an increase in acid secretion. A separate but smaller group has an increase in acid secretion with *H. pylori* with minimal inflammation in the body of the stomach and is at higher risk for duodenal ulceration. In this group, eradication of infection results in a fall in acid secretion, largely because of a fall in serum gastrin.^24^ As eradication of infection increases acid secretion in a substantial majority, the overall effect is an increase in the acid secretion that increases the risk of GERD. By extension, more prominent esophageal inflammation could increase the risk of Barrett's esophagus and the subsequent risk of adenocarcinoma. This hypothesis could explain the inverse relationship of *H. pylori* with Barrett's dysplasia and adenocarcinoma but other explanations are possible involving changes in the gastric microbiome or changes in the composition of refluxed fluid.

The epidemiologic evidence outlined above raises the possibility that the beneficial effects of *H. pylori* have been underestimated. Absence of infection has been associated with higher risks for obesity, IBD, and reflux oesophagitis and with the uncommon but important complications of reflux, namely Barrett's esophagus and esophageal adenocarcinoma. However, these risks need to be compared to the risk of gastric cancer and peptic ulceration. Policies that favor eradication regimens in infected individuals may well be appropriate in countries with a high burden of gastric cancer. However, similar policies in countries with a lower burden of gastric cancer may need to be re‐examined in the light of newer epidemiologic information. One day, perhaps, studies will examine the use of *H. pylori* for the treatment of human disease.
